# Performance and Safety of a Sodium Hyaluronate Tear Substitute with Polyethylene Glycol in Dry Eye Disease: A Multicenter, Investigator-Masked, Randomized, Noninferiority Trial

**DOI:** 10.1089/jop.2022.0048

**Published:** 2022-11-08

**Authors:** Marc Labetoulle, Bruno Mortemousque

**Affiliations:** ^1^ Service d'Ophtalmologie, Hôpital Bicêtre, APHP, Université Paris Sud, Le Kremlin-Bicêtre, France.; ^2^ IDMIT Infrastructure, CEA, Université Paris-Saclay, Inserm U1184, Fontenay-aux-Roses, France.; ^3^ Cabinet Ophtalmologie Foch, Bordeaux, France.

**Keywords:** dry eye, tear substitute, ocular lubricant, sodium hyaluronate, polyethylene glycol, clinical study

## Abstract

**Purpose:**

To compare the performance and safety of 2 tear substitutes containing sodium hyaluronate (SH); one containing 0.15% SH and polyethylene glycol (PEG) 8000, and the other containing 0.18% SH.

**Methods:**

*:* In this multicenter, randomized, investigator-masked, noninferiority trial, 83 patients with moderate or severe dry eye disease underwent a 2-week washout, and were then randomly assigned (1:1) to receive SH plus PEG tear substitute (*n* = 45) or comparator SH tear substitute (*n* = 38) 3–6 times daily for 3 months. The primary performance endpoint was the change from baseline in the ocular surface fluorescein staining (OSFS) score on day 28 in the per-protocol (PP) population, according to the 15-point Oxford Scheme, with a noninferiority margin of 2.

**Results:**

Both groups improved significantly in terms of signs and symptoms. Among the 78 patients without major protocol deviations (the PP population), the OSFS score decreased by 2.9 ± 2.0 on day 28 from 5.4 ± 1.3 at baseline in the SH plus PEG group and by 2.3 ± 2.2 from 5.2 ± 1.4 in the comparator group (95% confidence interval of the difference: −1.2 to 0.3), demonstrating noninferiority. On day 90, the improvement in OSFS scores was significantly greater in the SH plus PEG group (*P* = 0.0002). The safety profiles were satisfactory in both groups.

**Conclusion:**

SH plus PEG tear substitute was noninferior to SH tear substitute in the studied population and may provide additional benefits in the long term. ClinicalTrials.gov ID: NCT02975102.

## Introduction

Dry eye disease (DED; also known as keratoconjunctivitis sicca) is one of the most common ocular conditions and has a significant impact on public health. The estimated prevalence of DED ranges from 5% to 50% worldwide, depending on the study and diagnostic criteria. In some populations, when estimates are based on signs only, prevalence can reach 75%.^1^ DED is more commonly found in women than in men >40 years old, with a gradually increasing prevalence among both sexes over the age of 50.^2^ Moreover, the risk of DED has recently been rising in the younger population due to increasing use of electronic devices.^2,3^

The 2017 Dry Eye Workshop (DEWS) II report of the Tear Film and Ocular Surface Society (TFOS) redefines DED as “*a multifactorial disease of the ocular surface characterized by a loss of homeostasis of the tear film, and accompanied by ocular symptoms, in which tear film instability and hyperosmolarity, ocular surface inflammation and damage, and neurosensory abnormalities play etiological roles*.”^1^ Indeed, efficient tear production and turnover are essential for maintaining ocular surface health. The tear film, lacrimal glands, lacrimal outflow pathways, and corneal and conjunctival epithelia constitute a lacrimal functional unit (LFU).

Disease, damage, or a dysfunction of any LFU component can destabilize the tear film by causing alterations in the volume, composition, distribution, and/or clearance of the tear film, leading to local drying and hyperosmolarity of the exposed surface.^4^ This results in epithelial cell damage and induces a cascade of inflammatory events leading to the loss of mucin-producing cells and further drying, which enhances DED, creating a vicious circle.^5^ The condition can cause a wide range of symptoms, such as redness, stringy mucous, burning, and an itchy sensation. If left untreated, the disease can lead to chronic self-propelling inflammation, resulting in visual disturbance and tear film instability with potential damage to the ocular surface.^1^

The management of DED depends on the underlying cause(s) and the severity of symptoms. However, the treatment of this condition appears to pose unmet needs from most patients' and physicians' perspectives.^6^ Available treatments relieve symptoms, with tear substitutes being the mainstay. Tear substitutes are believed to act by replenishing the aqueous layer of the tear film and/or by restoring, and supporting the lipid layer to stabilize the tear film.^7^ Although tear substitutes are not meant to recapitulate the functions of natural tears, they have been shown to provide relief of symptoms and various signs in patients with DED.^7–10^

Despite a wide range of tear substitutes being available as first-line therapy in DED, management of dry eye patients remains challenging as treatment algorithms are not standardized according to disease progression due to variation in severity and character of this complex condition. In addition, relationship between signs and symptoms is not consistent from patient to patient.^7^ Thus, it is often useful to have a customized approach, proposing various tear substitutes with different properties and viscosities for patients to find the most adapted to their symptoms, activities, and environment. This approach can be more effective and increase treatment compliance than a rigid treatment strategy.^11^

Tear substitutes are buffered formulations containing electrolytes, surfactants, and one or more lubricants, such as sodium hyaluronate (SH) and polyethylene glycol (PEG).^12^ SH is a sodium salt of hyaluronic acid, a naturally occurring glycosaminoglycan present in the human eye structure and tear fluid. Its properties include water retention, slow release of moisture, tear film stabilization, lubrication, and mucomimetic behavior.^13–18^ In turn, PEG is an inert hydrophilic synthetic polymer, with a wide array of pharmaceutical applications.

As a demulcent, PEGs with molecular weights of ≥6,000 have lubricant properties.^19^ PEG can also interact with other polymers. Adding PEG 8000 to SH in a tear substitute increases its viscosity, strengthens the rheologic properties of SH, and enhances its distribution on the ocular surface, improving its wetting capability.^20,21^

Based on these add-on properties, the combination of 0.15% SH and PEG 8000 (SH-PEG) has been formulated. It was initially introduced on the European market in 2011, as a CE-marked, class IIb medical device. This combination has previously been studied in 15 patients with mild-to-moderate dry eye in an open trial. After 2 months of 3 times daily administration, improvement was reported for ocular inflammation, ocular surface damage, discomfort symptoms, tear film break-up time (TBUT), and osmolarity.^21^ As no randomized clinical trial was performed in moderate-to-severe dry eye during at least 3 months to show performance and safety, this trial was designed to provide such data.

For ethical reasons, placebo or saline used in a comparative group was not appropriate for these patients, and there is no consensus about the optimal comparator in such patients. Thus, a widespread tear substitute, with 0.18% SH, was selected as a comparator (C-SH). This comparator is considered as a pioneer SH tear substitute in Europe where it is widely marketed. Furthermore, it has demonstrated superiority to saline and noninferiority to carbomers in dry eye patients, and has been used in many clinical studies including several comparative similar studies demonstrating dry eye signs and symptoms improvement.^22–26^

## Methods

### Study design

This randomized, investigator-masked, noninferiority trial was performed in 18 centers (the CBL-101 study group), including 15 centers in France and 3 in Belgium. The study was conducted between March 2017 and October 2019, and involved 5 visits over a 15-week period. Selected patients underwent a 2-week washout period. Then, on day 0 (baseline), eligible patients were randomized (1:1), and follow-up visits took place on days 7, 28, and 90.

The study was conducted in accordance with the Declaration of Helsinki, the ISO 14155:2011 standard, and the Good Clinical Practice guidelines of the International Council for Harmonisation of Technical Requirements for Pharmaceuticals for Human Use, as well as with local regulations. The protocol, patient information, and consent form were approved by 2 independent ethics committees: one in Toulouse, France (Le Comité de Protection des Personnes Sud-Ouest et Outre-Mer II) and the other in Brussels, Belgium (Le Comité d'Ethique Hospital-facultaire ERASME-ULB).

### Study materials

All tear substitutes used in the study are CE-marked, class IIb medical devices provided in multidose bottles from commercial batches. The investigational product, SH-PEG (manufactured by Dr. Gerhard Mann, a Bausch + Lomb company), is marketed in European countries by local affiliates of Bausch + Lomb group under various names, including Elixya^®^, Artelac^®^ Rebalance, Biotrue^®^ Rebalance, and Biotrue reVITAL, and by Densmore Laboratoire as Vitadrop™. This formulation contains 0.15% SH with 0.5% PEG 8000 and Vitamin B12. The product contains a soft preservative, a stabilized chlorite complex (Oxyd^®^), which rapidly transforms into oxygen, water, and sodium chloride when administered.

The comparator product, C-SH, was Vismed^®^ Multi (Horus Pharma, manufactured by TRB Chemedica AG), which contains 0.18% SH and is preservative free. Both products also contain electrolytes that are naturally present in the tear film. In the treatment phase, patients instilled 1 drop of the assigned eye drops 3 to 6 times per day as needed for 90 days (± 10 days). During run-in treatment, preservative-free povidone 2% eye drops (Aqualarm^®^U.P., manufactured by Dr. Gerhard Mann) were used to exclude the use of different lubricants for a homogeneous study group at baseline.

The bottles of both products, although different, were relabeled with the same text (except for the randomization number) by an independent contract development and manufacturing organization responsible for issuing the randomization list in blocks of 4. To maintain single masking of the randomized products, the investigator had access only to sealed boxes identified by the randomization number.

### Study participants

The participants were required to be >18, to have at least one eye with signs of DED, defined as an ocular surface fluorescein staining (OSFS) score ≥4 and ≤9 (on the 15-point Oxford Scheme) and TFBUT ≤10 s, and to have a score ≥1 on a scale of 1–4 for at least 2 of the following 7 symptoms: dryness sensation, foreign body, burning, stinging, itching, blurred vision, and sensitivity to light. Patients needed to have used tear substitutes for at least 3 months before recruitment.

The main exclusion criteria were moderate or severe blepharitis, severe ocular dryness (OSFS score >9 and/or ocular surface disease), ocular surgery within 180 days, ocular trauma, nondry-eye ocular inflammation, ocular infection within 90 days before the study start, recent use of ocular or systemic therapy affecting dry eye (2 weeks to 3 months before the study, depending on the drug), pregnancy and breastfeeding.

### Study endpoints and assessments

The primary performance endpoint was the mean change in the OSFS score in the study eye according to the Oxford Scheme (0–15) between baseline and day 28. The study eye was the eligible eye with the highest OSFS score at baseline. If both eyes had the same OSFS score with TFBUT ≤10 s, the right eye was used as the study eye.

The secondary endpoints included the following: change from baseline in OSFS score on days 7 and 90; corneal, nasal conjunctival, and temporal conjunctival staining assessed independently (grades 0–5) at follow-up visits; change from baseline in the global dry eye symptom score (scales 0–28) and in TFBUT on days 28 and 90; change from baseline in unanesthetized Schirmer test on day 28; and the frequency of instillations (recorded in diaries). Paper diaries included completion instructions and checkboxes for number of instillations. In addition, investigators provided instructions and discussed them with patients.

Change from baseline in quality of life was assessed on day 90 using the validated Ocular Surface Disease-Quality of Life (OSD-QoL^®^) questionnaire including 28 items divided into 7 dimensions: daily activities, difficulties with work and handicap, giving up makeup, acknowledgment of the disease, acceptance of the disease, fear for the future, and emotional well-being. A global question: “How do you feel when considering your eye problems?” (included in the fear for the future dimension) was also evaluated separately. The converted score for each dimension ranges from 0 to 100, with a higher score reflecting a better quality of life.^27^

Safety evaluation included occurrence of adverse events (AEs) and assessment by the investigator of their relationship with study products according to product information and Good Clinical Practice training. Decimal visual acuity and slit lamp examination were also considered. Participants assessed eye drop tolerability including comfort upon instillation and blurred vision, as well as ease of use of the bottle by responding to a 4-point Likert scale questionnaire rated as strongly agree, agree, disagree, and strongly disagree.

### Statistical analysis

Using SAS^®^ version 9.4, a sample size of 33 evaluable patients per treatment group was calculated to provide at least 90% power to demonstrate noninferiority of SH-PEG tear substitute to C-SH tear substitute, assuming a noninferiority margin of 2 grades^23–26^ and a standard deviation of 2.5 on the primary endpoint, based on a similar published study.^23^ Considering potential dropouts and major protocol deviations (defined as impacting the primary endpoint), enrollment of 84 patients was planned.

The primary performance endpoint was analyzed using the baseline value as a covariate (SAS Mixed procedure). The 2-sided 95% confidence interval (CI) of the between-group difference in change from baseline was computed. Noninferiority would be demonstrated if the upper bound of the 95% CI was <2 in the per-protocol (PP) population, defined as all patients in the intention-to-treat (ITT) population without any major protocol deviations. Analysis in the ITT population (all randomized patients with at least one instillation and one postbaseline assessment) was also conducted.

The same covariance model was used to analyze secondary continuous endpoints in both PP and ITT populations. Categorical endpoints were analyzed using Fisher's exact test or Chi-square test, as appropriate. Safety endpoints were analyzed in the safety population, defined as all randomized patients with at least one instillation and follow-up information.

All statistical tests were performed at the significance level (α) of 0.05.

## Results

### Study population

**FIG. 1.**
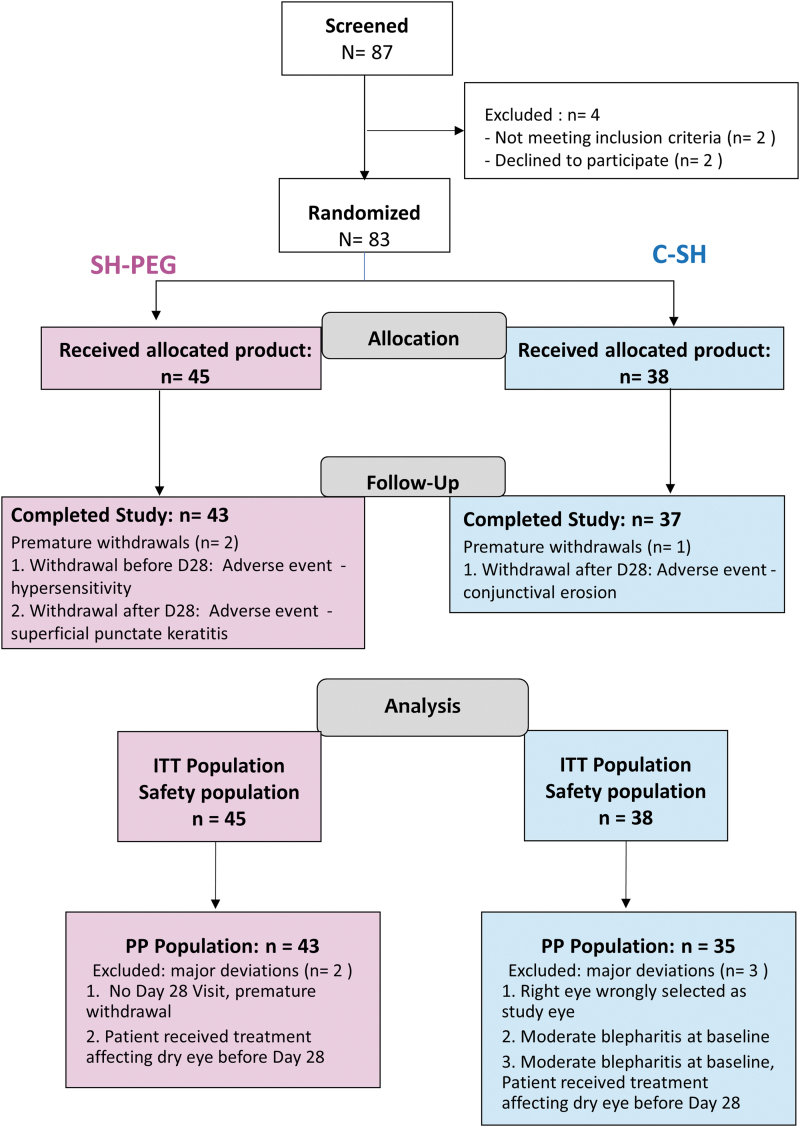
Patient flow diagram. C-SH, 0.18% sodium hyaluronate group; ITT, intention to treat; PP, per protocol; SH-PEG, 0.15% sodium hyaluronate-polyethylene glycol 8000 group.

Of the 87 screened patients, 4 were ineligible after washout; 2 patients withdrew consent (2–4 days after starting washout) and 2 did not meet inclusion criterion of OSFS score. Of the 83 eligible patients, 45 were randomized to the SH-PEG group and 38 to the C-SH group (Fig. 1); these patients constituted both the safety and ITT populations. Three of these patients withdrew prematurely due to AEs: 2 in the SH-PEG group and one in the comparator group. As only one of these 3 patients withdrew before the primary endpoint (day 28), only this patient of the 3 had a major deviation. There were 4 other patients with major deviation, and all 5 (Fig. 1) were excluded from the ITT population. Thus, the PP population consisted of 78 patients.

Demographic and baseline characteristics of the SH-PEG group were generally close to those of the C-SH group (Table 1). In brief, the mean age of combined participants was 62 ± 14 years, and most participants were female (76%). Even though there were more patients in the SH-PEG group with Sjögren's syndrome, the main dry eye etiology stated as “unknown” was reported for about two-thirds of patients in both groups. Demographic characteristics of the PP population reflected those of the ITT population.

**Table 1.** Demographic and Baseline Characteristics—Intention-to-Treat Population

	SH-PEG* n* = 45	C-SH* n* = 38	Total* N* = 83
Demographics			
Age (years)			
Mean ± SD	63.8 ± 14.0	60.6 ± 14.1	62.3 ± 14.0
Min; max	23.0; 90.0	30.0; 83.0	23.0; 90.0
Median	66.0	64.0	64.0
Gender			
Male	11 (24.4%)	9 (23.7%)	20 (24.1%)
Female	34 (75.6%)	29 (76.3%)	63 (75.9%)
Country			
France	39 (86.7%)	33 (86.8%)	72 (86.7%)
Belgium	6 (13.3%)	5 (13.2%)	11 (13.3%)
Dry eye history			
Age at onset (years)			
Mean ± SD	57.3 ± 14.2	54.3 ± 14.7	55.9 ± 14.4
Dry eye etiology			
Sjögren	7 (15.6%)	3 (7.9%)	10 (12.0%)
Other	8 (17.8%)	11 (28.9%)	19 (22.9%)
Unknown	30 (66.7%)	24 (63.2%)	54 (65.1%)
Dry eye duration (years)			
Mean ± SD	6.5 ± 5.6	6.3 ± 5.2	6.4 ± 5.4
Baseline characteristics			
Ocular surface fluorescein staining (0–15)			
Mean ± SD	5.4 ± 1.3	5.3 ± 1.5	5.3 ± 1.4
Global dry eye symptoms score (0–28)			
Mean ± SD	8.8 ± 2.7	8.2 ± 3.3	8.5 ± 3.0
Tear Film Break-up time (s)			
Mean ± SD	5.5 ± 1.6	6.0 ± 1.5	5.7 ± 1.6
Schirmer's test (mm/5 min)			
Mean ± SD	6.8 ± 3.7	7.4 ± 2.9	7.1 ± 3.3

C-SH, 0.18% sodium hyaluronate group; SD, standard deviation; SH-PEG, 0.15% sodium hyaluronate-polyethylene glycol 8000 group.

Patients' compliance to tear substitute instillation was calculated from the available diaries (92%). Self-reported compliance was high (at least 90%) and similar between groups.

### Performance results

#### Primary endpoint

On day 28, the OSFS score in the PP population decreased by 2.9 ± 2.0 from 5.4 ± 1.3 at baseline in the SH-PEG group and by 2.3 ± 2.2 from the baseline of 5.2 ± 1.4 in the C-SH group. The upper limit of the 95% CI of the adjusted difference (−0.4) between the change from baseline values of the 2 groups was 0.3, which was below the predefined noninferiority limit of 2 (Table 2). The results for the ITT population were consistent with those for the PP population, with a 95% CI of −1.3 to 0.3 for the difference between the groups.

**Table 2.** Performance Endpoints—Per-Protocol Population

	Day 0 (Baseline)	Day 28			Day 90		
	Mean ± SD	Mean ± SD	Adjusted change from baseline ± SE	Adjusted difference± SE [95% CI]/*P* value	Mean ± SD	Adjusted change from baseline ± SE	Adjusted difference ± SE [95% CI]/*P* value
Ocular surface fluorescein staining (0–15)							
SH-PEG	5.4 ± 1.3	2.5 ± 1.6	–2.9 ± 0.3	–0.4 ± 0.4 [−1.2 to 0.3]	1.4 ± 1.2	–4.0 ± 0.2	–1.4 ± 0.4 [−2.1 to −0.7] *P*^a^ = 0.0002
C-SH	5.2 ± 1.4	2.9 ± 1.8	–2.4 ± 0.3		2.8 ± 1.9	–2.6 ± 0.3	
Corneal staining (0–5)							
SH-PEG	1.8 ± 0.7	0.9 ± 0.8	–0.9 ± 0.1	–0.1 ± 0.2 [−0.5 to 0.2] *P* = 0.457	0.6 ± 0.6	–1.2 ± 0.1	–0.4 ± 0.2 [−0.7 to −0.1] *P*^a^ = 0.0186
C-SH	1.9 ± 0.8	1.1 ± 0.9	–0.8 ± 0.1		1.1 ± 0.9	–0.8 ± 0.1	
Nasal conjunctival staining (0–5)							
SH-PEG	1.8 ± 0.7	0.8 ± 0.7	–1.0 ± 0.1	–0.1 ± 0.2 [−0.5 to 0.2] *P* = 0.433	0.4 ± 0.6	–1.4 ± 0.1	–0.5 ± 0.2 [−0.8 to −0.2] *P*^a^ = 0.0011
C-SH	1.7 ± 0.7	0.9 ± 0.7	–0.9 ± 0.1		0.9 ± 0.7	–0.8 ± 0.1	
Temporal conjunctival staining (0–5)							
SH-PEG	1.8 ± 0.7	0.8 ± 0.6	–0.9 ± 0.1	–0.2 ± 0.1 [−0.5 to 0.1] *P* = 0.221	0.4 ± 0.6	–1.4 ± 0.1	–0.4 ± 0.2 [−0.7 to −0.1] *P*^a^ = 0.0050
C-SH	1.6 ± 0.5	0.9 ± 0.8	–0.8 ± 0.1		0.8 ± 0.7	–0.9 ± 0.1	
Global dry eye symptoms score (0–28)							
SH-PEG	8.8 ± 2.7	4.4 ± 3.0	*–*4.2 ± 0.4	–2.0 ± 0.6 [−3.1 to −0.9] *P*^a^ = 0.0008	3.4 ± 2.9	–5.1 ± 0.4	–2.0 ± 0.6 [−3.2 to −0.7] *P*^a^ = 0.0026
C-SH	7.7 ± 2.6	5.7 ± 2.8	*–*2.2 ± 0.4		5.0 ± 2.8	–3.1 ± 0.5	
TFBUT (s)							
SH-PEG	5.5 ± 1.6	6.7 ± 2.1	1.2 ± 0.2	0.4 ± 0.3 [−0.2 to 1.0] *P* = 0.231	7.2 ± 2.3	1.7 ± 0.2	0.9 ± 0.4 [0.2 to 1.6] *P*^a^ = 0.0135
C-SH	5.9 ± 1.5	6.7 ± 2.1	0.8 ± 0.2		6.7 ± 1.8	0.7 ± 0.3	
Schirmer's test (mm/5 min)							
SH-PEG	7.0 ± 3.8	8.9 ± 4.7	1.9 ± 0.5	0.4 ± 0.7 [−1.0 to 1.7] *P* = 0.575			
C-SH	7.2 ± 2.9	8.7 ± 4.0	1.5 ± 0.5				
OSD-QoL questionnaire global question							
SH-PEG	45.3 ± 21.9				64.9 ± 20.8	17.5 ± 3.0	8.4 ± 4.7 [*–*0.9 to 17.8] *P* = 0.077
C-SH	54.5 ± 22.6				60.7 ± 20.9	9.1 ± 3.5	
Frequency of instillations, daily							
SH-PEG					3.6 ± 0.9		*P*^a^ = 0.0389^b^
C-SH					3.9 ± 1.0		

^a^ Significant *P* values.

^b^ Mann–Whitney test.

CI, confidence interval; C-SH, 0.18% sodium hyaluronate group; OSD-QoL, Ocular Surface Disease Quality of Life; SD, standard deviation; SE, standard error; SH-PEG, 0.15% sodium hyaluronate-polyethylene glycol 8000 group; TFBUT, tear film break-up time.

#### Secondary endpoints

**FIG. 2.**
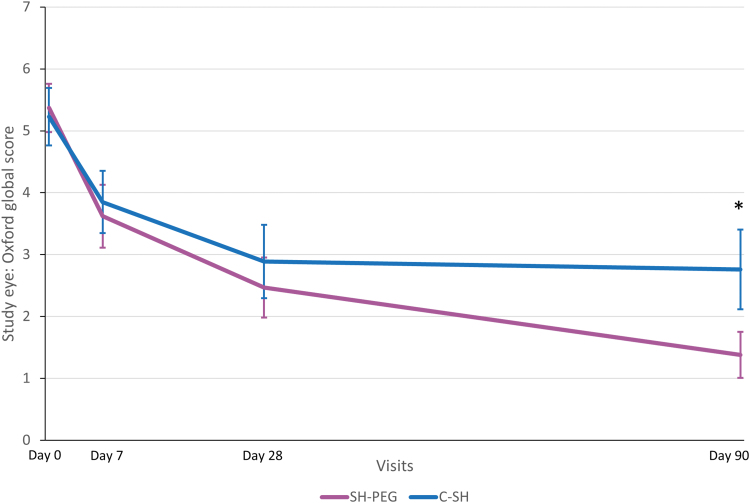
Ocular surface fluorescein staining score (mean ±95% confidence interval) at each visit in the per-protocol population. C-SH, 0.18% sodium hyaluronate group; SH-PEG, 0.15% sodium hyaluronate-polyethylene glycol 8000 group. *P = 0.0002.

**FIG. 3.**
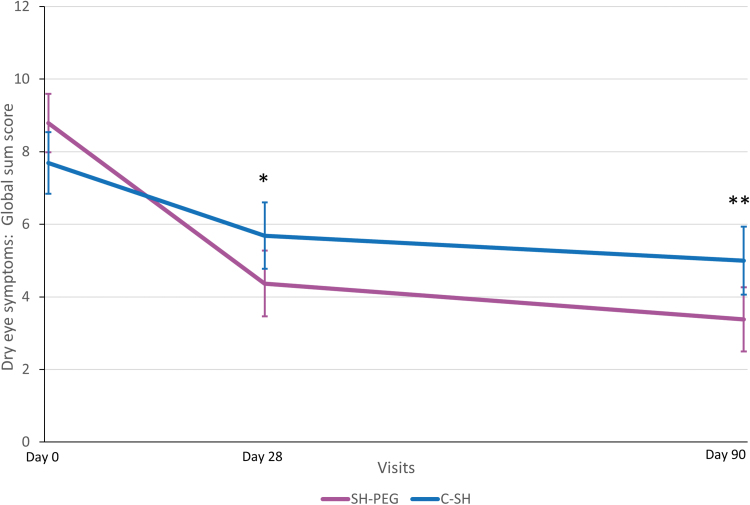
The global sum score of dry eye symptoms (mean ±95% confidence interval) in the per-protocol population. C-SH, 0.18% sodium hyaluronate group; SH-PEG, 0.15% sodium hyaluronate-polyethylene glycol 8000 group. *P = 0.0008; **P = 0.0026.

In the PP population, the change in OSFS scores from baseline to day 7 was similar between groups. However, a statistically significant difference was observed after 3 months, with better scores obtained by patients using SH-PEG (Table 2 and Fig. 2). When analyzing each of the subscores separately (corneal, nasal conjunctival, and temporal conjunctival staining), similar improvement was observed in both treatment groups on days 7 and 28; however, on day 90, patients using SH-PEG had significantly better scores than those in the C-SH group for each of the 3 ocular regions (Table 2). Similarly, the global sum score of dry eye symptoms had improved significantly more in the SH-PEG group than in the C-SH group at days 28 and 90 (Fig. 3).

The results of Schirmer's test conducted on day 28 showed similarities between the 2 groups, as did those of the TFBUT analysis. However, on day 90, the TFBUT was significantly longer in the SH-PEG group than in the C-SH group.

The frequency of daily instillations reported in the available dairies (39 in the SH-PEG group and 29 in the C-SH group) was similar between groups, with a mean of 3.6 ± 0.9 drops in the SH-PEG group and 3.9 ± 1.0 drops in the C-SH group. Median values were comparable with, respectively, 3.3 and 3.7 drops.

**FIG. 4.**
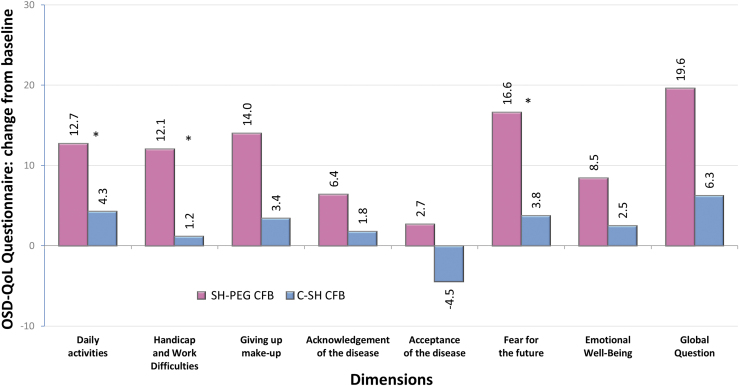
Quality of life assessments using the OSD-QoL^®^ questionnaire: change from baseline (day 0) on day 90 in the per-protocol population. C-SH, 0.18% sodium hyaluronate group; SH-PEG, 0.15% sodium hyaluronate-polyethylene glycol 8000 group; CFB, change from baseline; *P value ≤0.024.

Both groups had improvements in quality of life after 3 months of using the assigned tear substitute, with improvement reported for all the 7 dimensions covered by the OSD-QoL questionnaire in the SH-PEG group and for 6 of 7 dimensions in the C-SH group. For 3 of these parameters (daily activities, handicap and work difficulties, and fear for the future), improvements were significantly greater in the SH-PEG group (Fig. 4).

### Safety

No serious AEs were reported. Among the reported ocular events, eye irritation was the most frequent (2 patients in the SH-PEG group and one in the C-SH group), whereas other events were isolated cases. Overall, the incidence of ocular AEs was higher in the SH-PEG group than in the C-SH group (15.6% vs. 7.9%). However, the incidence of ocular events assessed by the investigator as being related to the investigational product was similar between groups (8.9% vs. 7.9%). In the SH-PEG group, these included eye irritation, corneal erosion, increase in dry eye, and hypersensitivity. In the C-SH group, these included corneal and conjunctival deposits, conjunctival erosion, and increase in dry eye. The reported nonocular events were also all isolated cases (5 patients overall) and assessed as unrelated to the study products.

AEs were the cause of premature withdrawal of 3 patients: 2 in the SH-PEG group (one case of hypersensitivity to the eye drops with severe symptoms of hyperemia, eye discharge, and increased lacrimation; and one case of moderate superficial punctate keratitis with eye irritation assessed as unlikely to be related to the product) and one patient in the C-SH group (severe conjunctival erosion assessed as possibly related to the product).

Visual acuity and slit-lamp assessments did not reveal any safety concerns, with results similar between groups.

### Tolerability and ease of use

At the end of study, the combined rating of “agree” and “strongly agree” that the eye drops were comfortable upon instillation was reported by at least 90% of patients in both groups. “Strongly agree” was rated by a higher percentage of patients in the SH-PEG group compared with the C-SH group (73% vs. 43%, respectively), whereas “agree” was rated by 21% in the SH-PEG group and 47% in the C-SH group. Less blurred vision was reported in the SH-PEG group (39% vs. 77%). Also, 69% of patients using SH-PEG strongly agreed that the bottle was easy to use compared with 31% in the comparator group (*P* = 0.002).

## Discussion

In our study, both tear substitutes demonstrated significant improvement in signs and symptoms of moderate-to-severe DED. In the primary analysis, SH-PEG was shown to be noninferior to C-SH in terms of change in OSFS score between baseline and day 28. Significant improvements in OSFS scores with both tear substitutes were also found on day 90, with a significantly greater improvement in the SH-PEG group. All secondary performance endpoints complemented the primary analyses. Although statistical significance might not always reflect clinical significance, the consistent improvements of these variables over time in the SH-PEG group suggest that addition of PEG 8000 to SH may bestow long-term benefits.

The results obtained from this study correspond to those obtained by other researchers. The results in the C-SH group of our study are consistent with those of another study, where carboxymethylcellulose was compared with Vismed (0.18% SH) in 82 patients over 3 months.^23^ In the Vismed (single unit dose) group, ocular surface staining (evaluated with corneal fluorescein staining and conjunctival lissamine green staining using the Oxford Scheme) had an adjusted decrease of 1.7 on day 35 and 2.5 on day 90 from a baseline value of 5.9. Concerning the combination SH-PEG product, in the previous study reported in 15 patients during 2 months,^21^ TFBUT improved significantly by a mean of 1.3 s, from 5.2 ± 2.3 to 6.5 ± 1.1 s. This is in accordance with our observations in the SH-PEG group, where we found an adjusted increase of 1.2 s after 1 month and 1.7 s after 3 months.

Our study revealed no safety concerns with either treatment. The difference in the preservative status of the 2 products was not expected to affect the results as the chlorite complex is a soft preservative, which when in contact with air (at time of instillation) turns immediately into oxygen, water, and sodium chloride, all naturally present on the eye surface. No significant increase in eye irritation or hyperemia was reported in the SH-PEG group. The safety profiles of both studied products were satisfactory. Furthermore, materiovigilance postmarketing 5-year data collection for the SH-PEG product showed a low frequency of AEs (0.0059%: data on file, Bausch+Lomb, France).

In most tear substitutes, the concentration of SH usually ranges from 0.1% to 0.3%. In a study evaluating different SH concentrations (0.1%, 0.15%, and 0.3%) in 131 patients administering the tear substitute 5–6 times a day for 12 weeks, no significant differences were reported between groups for fluorescein corneal staining, lissamine green conjunctival staining, and TBUT, although the Schirmer test results improved more in the 0.15% SH group.^28^

As a non-Newtonian fluid, the particular viscoelasticity of SH enables it to act differently during and between blinks. During blinks, it spreads easily over the surface of the cornea while between blinks, the solution is more viscous, slowing down the evaporation of water and extending its action on the ocular surface.^15–17^ SH solutions were also shown to adhere to mucins on the ocular surface, enabling them to form long-lasting ocular coatings,^18^ further prolonging their beneficial effects.

Both SH and PEG have been shown to improve symptoms in patients with DED when used separately in tear substitutes.^15,29^ The combination of 0.15% SH with PEG 8000 increases the viscoelastic properties of SH, improving its wetting capability,^20^ apparently without interfering with the optical quality of the lacrimal film, as shown by the lack of impact on vision in our study. Combining different polymers in a single formulation might have a synergistic effect, potentially providing enhanced benefits to the ocular surface.

The results of our study are in line with these enhanced properties, which could contribute to stabilizing the tear film, as well as to reducing ocular surface damage and symptoms, thus improving many characteristics of dry eye defined in the DEWS II report. By helping to keep the LFU functioning appropriately, the cascade of inflammatory events can be prevented, breaking the vicious circle of DED and preventing disease progression. The combination of SH-PEG can be an alternate opportunity within the available tear substitutes.

Although the study design considered the recommendations from the DEWS I report,^30^ using a randomized approach with 2 parallel groups, with an active comparator, several limitations should be discussed. The relatively short duration of the study can be a study limitation in DED. According to published studies, most of them are performed during 1–3 months, thus it appears that changes would be unlikely after 3 months for most treatments.^7^

Moreover, our study was investigator masked. Identical outer packaging was the only option available as the compared products were provided in the different original bottles (relabeled) to maintain their aseptic condition. When choosing the performance assessment methods, we considered standard practice in the management of DED. OSFS was chosen as the primary endpoint to assess the protective properties of the tear substitutes.

Ocular surface staining graded according to the 15-point Oxford Scheme is considered valuable for distinguishing changes,^31,32^ and has been used in several studies with significant improvement observed after 1 month of treatment with a SH-containing ophthalmic product.^21–26,33^ In our study, more patients were assigned to the SH-PEG group due to a decreased recruitment rate at some centers. Nevertheless, the demographics of the study population were in line with relevant epidemiologic studies, where most patients with DED are women aged >50.^2^

## Conclusions

Overall, both SH-PEG 8000 and C-SH tear substitutes performed well in patients with DED, with a satisfactory safety profile. The performance of SH-PEG was noninferior to that of C-SH after 28 days in the population studied. The secondary endpoint results supported these findings, and even suggest that, after 3 months, SH-PEG might outperform C-SH in reducing ocular surface staining and relieving dry eye symptoms, as well as in improving some aspects of quality of life. SH-PEG might therefore be a beneficial option in the management of moderate-to-severe DED. Further studies exploring the combination of SH and PEG in DED could provide a more detailed understanding of the possible synergic activity of these components and related benefits.

## Supplementary Material

Supplemental data

## Data Access Statement

The data that support the findings of this study are available from the corresponding author upon reasonable request. Relevant data are within the article and its Supplementary Table S1.
