# Supplementary material for: Performance and Safety of a Sodium Hyaluronate Tear Substitute with Polyethylene Glycol in Dry Eye Disease: A Multicenter, Investigator-Masked, Randomized, Noninferiority Trial
Source: J Ocul Pharmacol Ther. 2022 Nov 8;38(9):607–16. doi: 10.1089/jop.2022.0048 (PMC9700343; doi:10.1089/jop.2022.0048)
Supplement: Supplemental data [file Suppl_TableS1.docx]

**Table S1.** Demographic and baseline characteristics – per-protocol population.

|  |  | **SH-PEG n = 43** | **C-SH n = 35** | **Total  N = 78** |
| --- | --- | --- | --- | --- |
| **Demographics** | | | | |
| Age (years) | | | | |
|  | Mean ± SD | 64.2 ± 14.0 | 59.7 ± 14.3 | 62.2 ± 14.2 |
|  | Min ; max | 23.0 ; 90.0 | 30.0 ; 83.0 | 23.0 ; 90.0 |
|  | Median | 66.0 | 62.0 | 64.0 |
| Gender | | | | |
|  | Male | 11 (25.6%) | 8 (22.9%) | 19 (24.4%) |
|  | Female | 32 (74.4%) | 27 (77.1%) | 59 (75.6%) |
| Country | | | | |
|  | France | 37 (86.0%) | 31 (88.6%) | 68 (87.2%) |
|  | Belgium | 6 (14.0%) | 4 (11.4%) | 10 (12.8%) |
| **Dry eye history** | | | | |
| Age at onset (years) | | | | |
|  | Mean ± SD | 57.8 ± 14.2 | 53.4 ± 14.9 | 55.8 ± 14.6 |
| Dry eye etiology | | | | |
|  | Sjögren | 7 (16.3%) | 3 (8.6%) | 10 (12.8%) |
|  | Other | 8 (18.6%) | 10 (28.6%) | 18 (23.1%) |
|  | Unknown | 28 (65.1%) | 22 (62.9%) | 50 (64.1%) |
| Dry eye duration (years) | | | | |
|  | Mean ± SD | 6.4 ± 5.7 | 6.3 ± 5.4 | 6.4 ± 5.5 |
| **Baseline characteristics** | | | | |
| Ocular surface fluorescein staining *(0–15)* | | | | |
|  | Mean ± SD | 5.4 ± 1.3 | 5.2 ± 1.4 | 5.3 ± 1.3 |
| Global dry eye symptoms score *(0–28)* | | | | |
|  | Mean ± SD | 8.8 ± 2.7 | 7.7 ± 2.6 | 8.3 ± 2.7 |
| Tear Film Break-up-time *(seconds)* | | | | |
|  | Mean ± SD | 5.5 ± 1.6 | 5.9 ± 1.5 | 5.7 ± 1.6 |
| Schirmer’s test *(mm/5 min)* | | | | |
|  | Mean ± SD | 7.0 ± 3.8 | 7.2 ± 2.9 | 7.1 ± 3.3 |

C-SH: 0.18% sodium hyaluronate group; SD: standard deviation; SH-PEG: 0.15% sodium hyaluronate-polyethylene glycol 8000 group.
